# Uterine Fibroids in Black Women: A Race-Stratified Subgroup Analysis of Treatment Outcomes After Laparoscopic Radiofrequency Ablation

**DOI:** 10.1089/jwh.2020.9001

**Published:** 2022-04-19

**Authors:** Jay M. Berman, Linda Bradley, Soyini M. Hawkins, Barbara Levy

**Affiliations:** ^1^ Wayne State University Medical School, Detroit, Michigan, USA.; ^2^ Department of OB-GYN and Reproductive Biology, Cleveland Clinic, Cleveland, Ohio, USA.; ^3^ Fibroid and Pelvic Wellness Center of Georgia, Peachtree Corners, Georgia, USA.; ^4^ George Washington University School of Medicine and Health Sciences, Washington, District of Columbia, USA.

**Keywords:** uterine fibroids, quality of life, racial inequities, health care disparities, laparoscopic radiofrequency ablation, uterine leiomyomas

## Abstract

**Background:**

The disease and treatment burden of uterine fibroids (UF) in Black women is substantially greater compared with other racial groups, with higher rates of complications and poorer outcomes with both hysterectomy and myomectomy. The inequities in the access Black women have to minimally invasive routes of surgery contribute to their burden of illness. Laparoscopic radiofrequency ablation (LAP-RFA) is a minimally invasive, safe, and effective uterine-sparing treatment option.

**Methods:**

This subgroup analysis of the LAP-RFA Pivotal Trial stratified outcomes by race comparing White women (*n* = 28, 21%) versus Black women (*n* = 46, 34%).

**Results:**

At baseline, Black women had more fibroids on average (7.3 vs. 3.7; *P* ≤ 0.001), a greater symptom severity score (SSS) (*P* ≤ 0.001), and a lower health-related quality of life (HRQoL) score (*P* = 0.005) than White women. At 36 months post-treatment with LAP-RFA, the statistical differences that existed with baseline SSS and HRQoL score were eliminated between Black and White women. Menstrual blood loss (140.1 mL vs. 127.02 mL; *P* = 0.44) and mean fibroid volume reduction (47.5 cm^3^ vs. 36.0 cm^3^; *P* = 0.17) were similar between Black and White women at 12 months. Although not statistically significant, the intraoperative total blood loss and uterine blood loss was lower in Black women than White women, despite greater operative time (160 minutes vs. 137 minutes; *P* = 0.09).

**Conclusions:**

These results are promising in providing an alternative uterine-sparing option for Black women and may help to provide a minimally invasive option that can address some of the racial inequities in care for Black women with UF.

## Introduction

As one of the most common forms of benign pelvic tumors,^1,2^ uterine fibroids (UF) have a broad range of prevalence estimates (depending on study population and diagnostic methodology) ranging between 4.5% and 68.6% in the United States population.^3^ The incidence of UF corresponds to increasing age—affecting an estimated 80% of women by age 50.^4^ Compared with other racial groups, there is a substantial disparity for Black women—in the United States, Black women have a threefold greater incidence and relative risk of fibroids, and an earlier age of onset compared with White women.^5^ Black women are two to three times more likely to develop UF and have a higher rate of comorbidities, such as obesity, diabetes, and hypertension.^6^ While individual variations exist based on specific risk factors, according to one analysis (conducted by Innovative Analytics), women who were identified by race as Black were observed to have both larger and a greater number of fibroids overall, compared with counterparts who were identified as White.^7^

Racial disparities are also evident in the treatment of UF—Black women are 2.4 times more likely to undergo a hysterectomy and have a 6.8-fold increased risk of undergoing myomectomies.^8^ At the time of hysterectomy, Black women have higher uterine weights, more fibroids, a higher likelihood of preoperative anemia, blood transfusion and severe pelvic pain. These disparities have strong regional associations, as they are more pronounced for premenopausal Black women in the southern United States.^8,15^

Recent literature has underscored the increased morbidity associated with both myomectomy and hysterectomy for Black women, with significant inequities in the rates of complications and poorer outcomes for both procedures in this population.^5,6,8^ UF are the leading cause of benign hysterectomies in the Unites States and there are proportionately more hysterectomies performed on Black women compared with White women.^1,2,6^ Black women experience higher rates of open hysterectomy for UF treatment, with more major and minor postoperative complications, and have a decreased likelihood of receiving minimally invasive hysterectomies when compared with White women.^6,9,10^ Hysterectomy for multiple and large UF also poses a significant morbidity risk.^10^ Compared with White women, Black women who undergo hysterectomies are four times as likely to develop complications and nearly three times as likely to be hospitalized, with a higher likelihood of mortality.^11^

Myomectomy is currently a uterine-sparing standard of care in gynecologic surgery, however, performing multiple myomectomies for a large number of fibroids can be a complex procedure requiring significant surgical expertise and skill, with risk for considerable blood loss, transfusion, and complications.^1,9^ Black women are 50% more likely to experience morbidity due to blood transfusion, surgical site infection, wound dehiscence, and sepsis after an abdominal myomectomy compared with White women.^10^ An analysis of a large multicenter database showed that Black women undergoing myomectomy were 1.6 times as likely to suffer a major complication and 2.27 times as likely to undergo a blood transfusion compared with White women.^9^

The inequities in the access that Black women have to minimally invasive routes of surgery contribute to their burden of illness.^10,11^ The burden of disease and the lack of low-risk uterine-sparing options and access may lead many Black women to unwanted hysterectomies for symptom relief. There is an unmet need for uterine-sparing treatment options that are both safe and effective and demonstrate equally low reintervention rates when compared with myomectomy. While this unmet need exists across all women, additional treatment options can provide a valuable opportunity to address racial inequities in care for Black women who are suffering from UF. The laparoscopic radiofrequency ablation (LAP-RFA) procedure, a minimally invasive ultrasound-guided treatment for symptomatic UF, has been shown to reduce the risk for transfusion, create equivalent symptom relief at 3 years, and offers an important option in the treatment armamentarium.^1,12^

The first-year results of the LAP-RFA pivotal trial were reported by Chudnoff et al.,^13^ and demonstrated evidence of significant decrease in menstrual blood loss and a nearly 50% reduction in UF volume at 1 year, compared with baseline. Furthermore, patient-reported outcomes (PROs), such as the Uterine FIbroid Symptom and Quality of Life (UFS-QoL) score, were improved at 3 months.^13^ The 2-year outcomes for the same LAP-RFA trial were then reported in Guido et al.,^16^ and demonstrated the durability of the results. Finally, the 3-year outcomes were reported by Berman et al.,^14^ and verified the sustained relief across all endpoints at 36 months following the LAP-RFA procedure, and showed continued significant improvement across both PRO scales.^14^ The following is a subgroup analysis of the LAP-RFA pivotal trial; a study conducted on women with symptomatic UF who were treated with LAP-RFA and evaluated for outcomes for 3 years.^14^ This subgroup analysis compared the short and long-term outcomes of treatment with LAP-RFA specifically stratified by race among this cohort. The primary outcome was the change in patient-reported outcomes over the course of the study.

## Methods

This study was a subgroup analysis of the LAP-RFA Pivotal Trial (NCT00874029). Methods of the pivotal clinical trial were previously described by Berman et al.^14^ Briefly, the multicenter, international trial consisted of 137 women with symptomatic UF, who met inclusion criteria, all of whom received LAP-RFA. Of this population, 135 were included in the full analysis with 3-year outcomes data.^14^ Patients were monitored over a 3-year period for menstrual blood loss, PROs, myoma volume reduction, and adverse events.^14^ For this subgroup analysis, the patient population was stratified by race, as patient groups analyzed included Black and White women (*n* = 74). Other racial groups within the study population included Asian, Hispanic, and Hispanic indigenous or Caribbean. The focus of this subgroup analysis was a comparison between patients who identified as Black and White, therefore, other races were not considered and included within this analysis. The protocol was deemed to be exempt from Institutional Review Board review and approval as it is secondary research for which consent is not required.^17^

Outcome measures included menstrual blood loss, procedure response, mean fibroid volume reduction, intraoperative outcomes, and validated PROs. Menstrual blood loss was measured at baseline and at 12 months post-procedure to determine a change in blood loss over the 12-month period using alkaline hematin analysis of subjects' catamenial products (pads, tampons, and liners). Intraoperative outcomes included blood loss (both total and uterine only blood loss), and procedure time from incision to skin closure. Blood loss reported with “≤xx” were treated as “xx mL” for analysis (*e.g.*, “≤50” was assumed to be 50 mL). Procedure response metrics included PRO outcomes following the procedure at 36 months and the mean fibroid volume reduction at 12 months, as measured by magnetic resonance imaging. PROs were assessed from written responses to the UFS-QOL questionnaire from patients at baseline and each follow-up visit (3, 6, 12, 24, and 36 months), specifically focused on the Symptom Severity Score (SSS) scale and Health-Related Quality of Life (HRQoL) scale. In this subgroup analysis, SSS and HRQoL scores were reported at baseline, 12, 24, and 36 months to compare the change in score over the 3-year period in Black women and White women. Additional health resource utilization and burden parameters, including operative time, the number of reinterventions post-procedure at 36 months, and missed workdays were also observed.

Statistical analysis was performed using Python's NumPy and SciPy packages. Continuous and discrete variables for these analyses were summarized using descriptive statistics, including mean, standard deviation, difference, and 95% confidence interval. A p-value of 0.05 was considered statistically significant. The p-values for race-based comparisons were based on a *t*-test or a Wilcoxon test if data were non-normally distributed. All statistical analyses were performed using SAS version 9.4 (SAS Institute, Inc., Cary, NC).

## Results

**FIG. 1.**
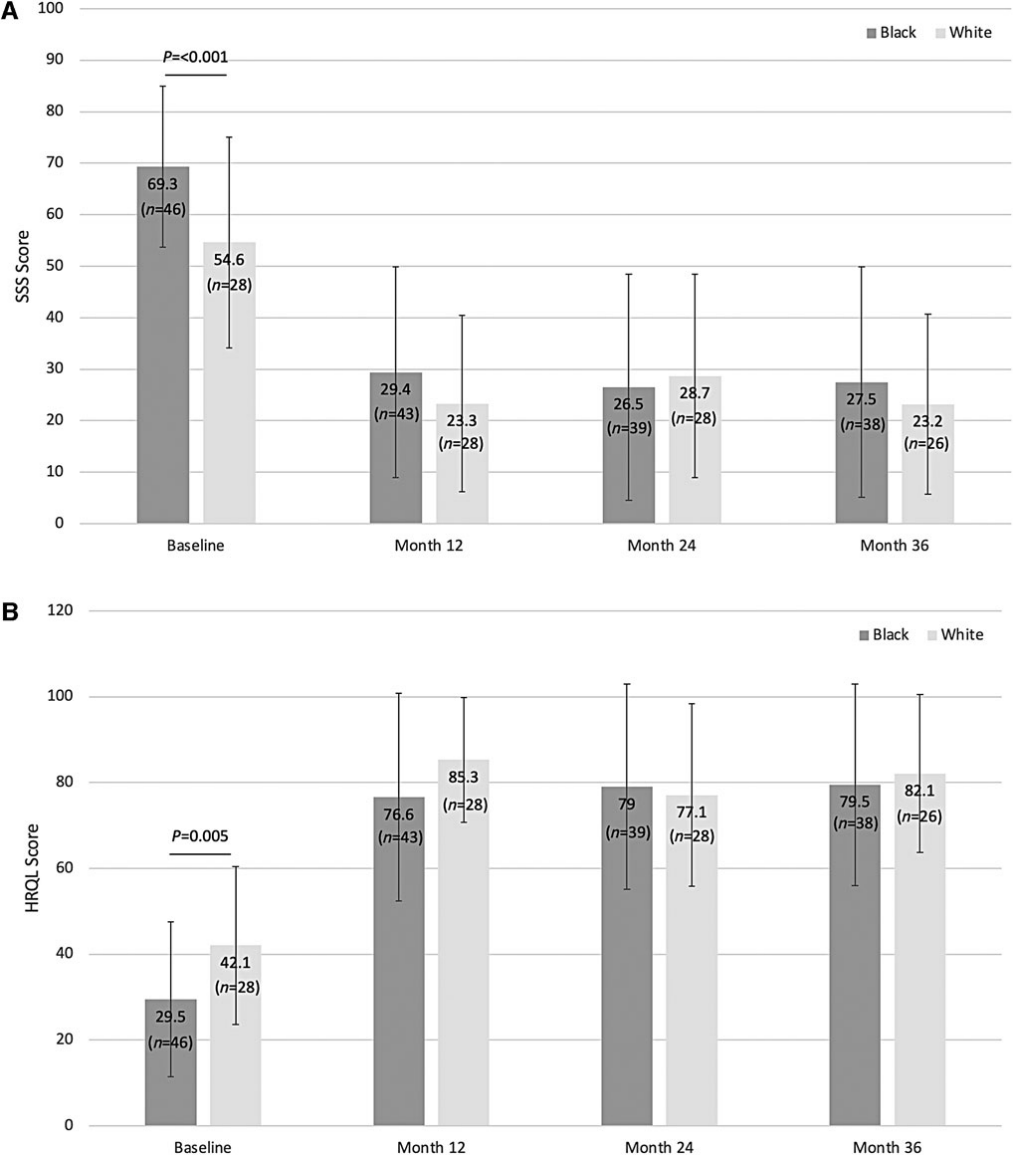
Quality of life metrics. Data are presented as mean ± SD. *p*-values are based on a *t*-test or Wilcoxon test. **(A)** SSS score from baseline. The difference (Black − White) [95% confidence intervals] were 14.7 [6.3–23.1] at baseline, 6.1 [−3.2 to 15.4] at 12 months, −2.1 [−12.6 to 8.3] at 24 months, and 4.4 [−6.1 to 14.8] at 36 months. **(B)** HRQoL score from baseline. The difference (Black − White) [95% confidence intervals] were −12.6 [−21.2 to −3.9] at baseline, −8.6 [−18.8 to 1.5] at 12 months, 2.0 [−9.3 to 13.3] at 24 months, and −2.6 [−13.5 to 8.4] at 36 months. HRQoL, health-related quality of life; SSS, symptom severity score.

Among the patients analyzed from the LAP-RFA Pivotal Trial (*n* = 135), 74 were included in this subgroup analysis of White women (*n* = 28) and Black women (*n* = 46). All three measurements taken at baseline showed a significantly higher disease burden for Black women compared with White women (Table 1). Two validated, qualitative surveys were used to assess outcomes and quality of life. The baseline SSS score was significantly greater for Black women compared with White women (*P* ≤ 0.001) indicating more symptoms. The baseline HRQoL score was significantly lower for Black women compared with White women (*P* = 0.005), indicating a lower quality of life (Fig. 1). Of the 74 patients included in this subgroup analysis, a total of 8 patients were lost to follow-up or withdrew, or had pregnancies over the entire 3-year study period. Of the subjects who were lost to follow-up or withdrew from the study, five were Black and one was White; one additional subject in the Black group had a missing questionnaire during year 2 analysis. Pregnancy occurred in one Black subject within the subgroup analysis over the 3-year period.

**Table 1.** Baseline Demographics

Metric	Black race (*n* = 46)	White race (*n* = 28)	Difference (Black − White) (95% CI)	*p*-value^a^
Age (years), mean ± SD	42.6 ± 4.2	44.7 ± 4.3	−2.1 (−4.1 to −0.1)	0.04
Height (cm), mean ± SD	166.5 ± 7.5	163.5 ± 7.8	3.0 (−0.7 to 6.6)	0.11
Weight (kg), mean ± SD	89.5 ± 19.9	76.3 ± 15.9	13.2 (4.4 to 22.1)	0.004
Body mass index, mean ± SD	32.2 ± 6.3	28.4 ± 4.8	3.9 (1.1 to 6.6)	0.007
No. of demonstrable fibroids through MRI, mean ± SD	5.6 ± 4.0	3.6 ± 2.0	2.0 (0.7 to 3.4)	0.005
Baseline SSS score, mean ± SD	69.3 ± 15.6	54.6 ± 20.5	14.7 (6.3 to 23.1)	≤0.001
Baseline HRQoL score, mean ± SD	29.5 ± 18.0	42.1 ± 18.4	−12.6 (−21.2 to −3.9)	0.005

^a^ *p*-values are based on a *t*-test.

CI, confidence interval; MRI, magnetic resonance imaging; HRQoL, health-related quality of life; SD, standard deviation; SSS, symptom severity score.

Black women had an average of 7.3 fibroids treated compared with 3.7 fibroids for White women (*P* ≤ 0.001). The procedure time was longer for Black women compared with White women (160 minutes vs. 137 minutes, respectively), although this difference was not significant (*P* = 0.09). Intraoperative total blood loss and uterine blood loss was lower in Black women than White women, despite greater operative time and a larger number of fibroids treated. This was not a statistically significant difference (Table 2).

**Table 2.** Intraoperative Results

Metric	Black race (*n* = 46)	White race (*n* = 28)	Difference (Black − White) (95% CI)	*p*-value^a^
No. of treated fibroids	7.3 ± 5.2	3.7 ± 3.3	3.6 (1.6 to 5.5)	≤0.001
Intraoperative total blood loss (mL)^b^	35.2 ± 25.5	37.8 ± 24.5	−2.5 (−14.6 to 9.5)	0.61
Intraoperative total uterine blood loss (mL)^b^	29.0 ± 21.1	37.3 ± 25.2	−8.3 (−20.0 to 3.4)	0.20
Operating room time (minutes)	159.6 ± 51.6	136.6 ± 63.4	23.0 (−3.9 to 49.9)	0.09

^a^ *p*-values are based on a *t*-test or Wilcoxon test.

^b^ Blood loss reported with “≤xx” were treated as “xx mL” for analysis (*e.g.*, “≤50” was assumed to be “50 mL”).

At 36 months post-treatment with LAP-RFA, the statistical differences that had existed in PROs at baseline were eliminated. There were no significant differences in SSS and HRQoL scores between Black and White women at 12, 24, or 36 months (Fig. 1). Menstrual blood loss at baseline, 3, 6, and 12 months was similar for both groups. There was a 30% and 32% reduction in menstrual blood loss by 3 months and a 50% and 53% reduction by 12 months, respectively for Black and White women (Table 3). Mean fibroid volume reduction at 12 months was similar between groups, as well (47.5 cm^3^ vs. 36 cm^3^), respectively for Black and White women; *P* = 0.17) (Table 3). The number of reinterventions by 36 months was identical between both groups with 3 and 3 occurring in the Black and White groups, respectively. These reinterventions occurred between 14- and 23 months post-procedure. In year 2, there were four reinterventions that included three hysterectomies (two Black women, and one White woman) and one hysteroscopic myomectomy (in a Black woman). In year 3, both reinterventions were hysterectomies in White women.

**Table 3.** Outcomes Post Procedure

Metric	Black race	White race	Difference (Black − White) (95% CI)	*p*-value^a^
Menstrual blood loss (mL), mean ± SD				
Baseline	280.6 ± 90.7 (*n* = 46)	267.6 ± 83.2 (*n* = 28)	13.0 (−29.0 to 55.0)	0.64
3 Months	198.0 ± 215.2 (*n* = 32)	180.9 ± 103.9 (*n* = 23)	17.1 (−80.3 to 114.5)	0.75
6 Months	148 ± 90.0 (*n* = 22)	140 ± 83.1 (*n* = 13)	8.9 (−53.4 to 71.2)	0.99
12 Months	140.1 ± 63.8 (*n* = 9)	127.0 ± 121.7 (*n* = 7)	13.0 (−87.7 to 113.7)	0.44
Fibroid volume reduction at 12 months (cm^3^), mean ± SD	47.5 ± 52.1 (*n* = 38)	36.0 ± 49.6 (*n* = 27)	11.4 (−14.3 to 37.1)	0.17

Data are presented as mean ± SD.

^a^ *p*-values are based on a *t*-test or Wilcoxon test.

## Discussion

This is the first study to analyze LAP-RFA outcomes stratified by race. Uterine-sparing options that are safe, and as effective as myomectomy that demonstrate equally low reintervention rates are critically needed. The results of this study indicate that LAP-RFA is an effective minimally invasive treatment option for Black women with UF and has the potential to specifically address racial inequities in care received by this group. In this study, the baseline disease burden experienced by Black women was higher than White women with higher number of treated fibroids, lower reported quality of life and higher symptom severity. These findings are consistent with other lines of evidence in published literature.^5,6,18,19^ Kjerulff et al., a comparative study of racial differences in UF, reported that Black women were likely to have seven or more fibroids that were larger with a younger age at diagnosis and more severe symptoms compared with their White counterparts.^18^ A similar difference was shown in Moorman et al., with Black and White women undergoing postmenopausal hysterectomy having 9.9 and 4.5 mean number of fibroids detected by ultrasound, respectively, with a significant difference between racial groups.^19^

According to the Society for Women's Health Research and the United States Food and Drug Administration Office of Women's Health, sex- and race-based disparities extend beyond health care access. Black women are underrepresented in clinical trials due to limitations in access and enrollment, a mistrust of minoritized individuals in research, and a history of scientific racism that has shaped how minority populations respond to research recruitment.^20^ Within the United States, it is estimated that 13% of the population are Black women,^21^ however, typically Black women only comprise 5% of clinical trial participants.^20^ Efforts by regulators to improve disparities have not been effective in improving representation in clinical trials.^22,23^ Within the LAP-RFA pivotal trial population, it was observed that Black women comprised 34% of the total study population. Based on the clinical experience of the authors, the demographic representation is a key differentiator of this data set in comparison to typical study populations.

As previously reported by Berman et al., the entire LAP-RFA pivotal trial study population experienced a clinically significant improvement in both PRO scales from baseline to 36 months.^14^ One year outcomes data described a significant decrease in menstrual blood loss and almost a 50% reduction in UF volume at 1 year, compared with baseline.^13^ The UFS-QoL score and SSS both improved at 3 months and maintained this improvement until the end of the study period at 36 months.^14^

In this subgroup analysis, at baseline, there were statistically significant differences in multiple outcomes with Black women experiencing more symptoms and lower quality of life. By 36 months post-procedure, these differences were eliminated—these data suggest that LAP-RFA was as effective or more effective for Black women compared with White women. The differences in baseline PROs were eliminated by the LAP-RFA procedure. Although Black women had more fibroids, a similar volume of menstrual blood loss at 12 months was in both Black and White women. Additionally, despite a longer operative time for Black women, the observations of total and uterine intraoperative blood loss were similar between both Black and White women. Overall, these results indicate that the LAP-RFA procedure had a greater impact on recovery and health outcomes for Black women compared with White women. These promising LAP-RFA outcomes are compelling for Black women, given their risk of more invasive surgical interventions, higher clinical risk, and morbidity.^5,6,8,9,11^

As noted in previous studies, a primary benefit of LAP-RFA is the inherent safety and rapid recovery.^24,25^ As previously concluded in a meta-analysis by Bradley et al., multiple delivery approaches of RFA technology have been used more frequently in recent years.^24^ It was noted that RFA, across delivery approaches (including laparoscopic), consistently showed sustained fibroid volume reduction, along with significant improvements in HRQoL and SSS scores and favorable rates of surgical reintervention. LAP-RFA is unique in its ability to use laparoscopic intra-abdominal ultrasound guidance to treat symptomatic UF. Beyond the previously reported 1-, 2-, and 3-year outcomes of the pivotal trial,^13,14,16^ two additional studies conducted in Canada and Germany compared LAP-RFA with myomectomy. Rattray et al. concluded that LAP-RFA was associated with significantly lower intraoperative blood loss, shorter procedure and hospitalization times, reduced health care resource utilization, and a quicker return to work through 3 months following treatment.^26^ Brucker et al. described that LAP-RFA resulted in the treatment of more fibroids, a significantly shorter hospital stay, and less intraoperative blood loss when compared with laparoscopic myomectomy.^27^ This analysis builds on past publications by confirming the outcomes for LAP-RFA, including less intraoperative blood loss, fewer serious adverse events, fewer complications, and fewer missed days of work when compared with published outcomes for myomectomy or hysterectomy.^1,9,28–35^ Furthermore, based on this subgroup analysis, LAP-RFA resulted in equivalent safety outcomes and rapid recovery for Black women compared with White women. LAP-RFA warrants further study of outcomes based on race to ensure the findings are robust and to better inform patients in clinical practice.

### Limitations

This study was a subgroup analysis of a larger study with a relatively small sample size, indicating a limited ability to draw widespread conclusions that apply to the general population. Further analysis and stratification of data in larger populations is warranted to draw such conclusions. Multiplicity adjustments were not applied in this subgroup analysis; therefore, the results require cautious interpretation and may potentially represent chance findings.

## Conclusions

Despite a higher baseline burden of disease, LAP-RFA was equally or more effective for Black women when compared with White women, resulting in equalizing patient-reported quality of life indices by 36 months post-procedure. These results are consistent with the clinical outcomes observed by the authors and are promising in providing an alternative uterine-sparing option for Black women.

The treatment and management of UF requires a patient-centric approach, focused on individual preference for uterine preservation. LAP-RFA may help close the gap by providing gynecologic surgeons with an additional minimally invasive treatment option that can safely and effectively treat a large number of fibroids. Furthermore, there are widespread racial and socioeconomic considerations to the treatment and management of UF. Although addressing racial inequities in care on a broader scale requires a multipronged approach, Black women, who present with more severe UF, can benefit from minimally invasive options as an alternative to hysterectomy.

Future research may include a registry following women undergoing uterine preserving surgical intervention for UF, which can validate the results of this study. Collecting structured data and stratifying by race will provide a more robust platform for understanding disparities in surgical fibroid management and clinical outcomes. Although it is unrealistic to expect further surgical randomized controlled trials, leveraging real-world evidence in combination with this study, and others, will help solidify our understanding of disparities in both treatment and outcomes. Additional studies can benefit from including a comparison of all uterine-sparing treatment options, stratification of treatment outcomes by socioeconomic status within each race, and analysis of access to care by racial groups to understand the complexities of these equity issues.
